# Research on double camouflage encryption mechanism of QR code based on UAV landing scenario

**DOI:** 10.1038/s41598-023-49104-2

**Published:** 2023-12-08

**Authors:** Hualong Ye, Tongxu Xu

**Affiliations:** School of Electrical and Information Engineering, Changshu Institute of Technology, Suzhou, 215500 China

**Keywords:** Optics and photonics, Physics

## Abstract

Usually, the landing area of the drone is presented with QR code images, so it is crucial to ensure the information security of the landing area and prevent it from being occupied by other users. This paper proposes a double camouflage encryption method of QR code based on UAV landing scenario. For the QR code image required for UAV landing, the private key and carrier image are used to complete double camouflage encryption, and then the public key is modulated according to the principle of ghost imaging to obtain the ciphertext. After receiving the ciphertext, the receiver first decrypts the camouflage image according to the public key, and then decrypts the QR code image using the private key. The UAV receives ciphertext information through the detector, for non-users, the correct QR code image cannot be decrypted through the wrong key. Even if the eavesdropper obtains the public key information, they can only decrypt the camouflage image and cannot land. For our users, the public key and the double private key can decrypt the correct QR code image for landing. This encryption method can effectively decrypt the image at non-full sampling rate, while also resisting the external noise attack, and has high security.

## Introduction

With the development of science and technology, an important measure to ensure the security of information transmission is to adopt information encryption technology. Information encryption is the use of some technical means to encode encrypted plaintext information to generate an unrecognizable ciphertext (encryption process). If the recipient holds the correct key, the ciphertext message can be restored (decryption process). No one can restore the ciphertext message without the correct key. Optical encryption is one of the mainstream information encryption technologies. Compared with traditional encryption methods, optical encryption has attracted more and more attention due to its advantages of high speed, parallelism and low cost.

In the optical field, Ghost Imaging (GI) technology can generate the image of an object on the optical path that does not contain an object, which can solve problems of conventional imaging failure or difficulty in solving. Moreover, the ghost imaging optical path is simple and requires low equipment requirements, making it easy to combine with other image systems, making it a promising application scenario and capable of playing a unique role in fields such as medical, military, remote sensing, encryption, radar, etc.^1,2^. With the in-depth research and exploration of ghost imaging, GI technology is gradually mature and gradually applied to the field of image encryption. For optical image encryption, the ghost imaging encryption scheme has been developed rapidly since it was proposed. In 2013, Kong et al. used the associated position of the object signal and the reference signal as the key to achieve optical encryption^3^. In 2015, Zhao et al. proposed an encryption scheme based on fast response code and ghost imaging, plaintext information is first encoded into fast response code and then encrypted by ghost imaging technology, this scheme has good anti-eavesdropping performance^4^. In 2016, Yuan et al. analyzed the vulnerabilities of ghost imaging encryption schemes and proposed a supply scheme for ghost imaging^5^. In 2019, Sui et al. introduced custom data containers into the computational ghost imaging encryption scheme, thus solving the problem of the inherent linearity of computational ghost imaging and the fuzzy reconstructed image caused by the imaging mechanism^6^. In order to further increase the amount of information in encryption and improve the efficiency of the ghost imaging encryption scheme, in 2016, Wu et al. took the lead in implementing a multi-image encryption scheme based on ghost imaging by using the location multiplexing method^7^. In the same year, Meng et al. introduced Logistic mapping and coordinate sampling into the ghost imaging encryption scheme, which realized multi-image encryption while reducing the amount of ciphertext data^8^. In 2018, Xu et al. studied a compressed sensing ghost imaging multi-image encryption scheme based on threshold secret sharing and line scanning^9^. In 2019, Zhang et al. performed Fourier transform on the image and extracted the central part to realize information compression, and then realized multi-image encryption by means of spatial multiplexing^10^. In 2020, Quan et al. proposed a novel ghost imaging authentication method, which transforms into a one-dimensional vector through block operation and transmits it together as a public key and ciphertext^11^. In 2022, Patra A et al. proposed a new method using phase grating to multiplex as well as encrypting 32 cross-sectional CT scan images (slices) in a single canvas for optimization of storage space and improvement of security^12^. In 2023, Yuan et al. proposed a multi-user optical encryption scheme based on ghost imaging, in which a common ciphertext is disclosed to all users, this scheme not only reduces the transmission amount of key data, but also improves the quality of decrypted images^13^. With the continuous progress of society, the research of ghost imaging in the field of optical encryption is becoming more and more mature^14,15^.

In recent years, UAV remote sensing has many advantages, such as low cost, high spatio-temporal resolution, little impact by weather environment, etc., so that it has a unique advantage in ground object recognition ability, and information acquisition is more rapid and detailed, which greatly improves the efficiency of information acquisition. It effectively makes up for the constraints of satellite aerial remote sensing system due to low image resolution, large impact of weather environment, long revisit period and other factors, and has become one of the research hotspots of medium and small-scale remote sensing application^16^. Autonomous landing^17^ has always been one of the most important aspects of UAV flight. Due to the problems of low precision, large error and high cost in traditional navigation methods, while autonomous landing methods with higher accuracy and smaller error are required in plant protection, logistics, military and other fields, the combination of image processing technology^18^ and UAV technology has become a new trend. In the field of UAV landing technology, there are relatively complete schemes for static landing sites, and the technology is also becoming mature^19–21^. In 2020, Yang et al.^22^ designed a machine vision system to assist the automatic landing of UAV and detect the landing site on the horizontal ground. In 2021, Lin et al.^23^ realized the hovering and landing of the UAV on the mobile platform by combining the artificial ground identification April Tag with the visual guidance and tracking algorithm of the UAV. In the UAV landing scene, the selection of landing area sign image is very important. QR codes are most commonly used in drone landing areas. QR codes have the characteristics of high data storage capacity, strong fault tolerance, multi-angle high-speed recognition, etc., and have been extensively studied by relevant workers in recent years^24–26^. However, in the field of UAV landing, the landing point identification image is highly likely to be cracked by eavesdropping drones, thus occupying the landing area of the correct receiver.

Based on this, this paper proposes a double camouflage encryption method of QR code based on the UAV landing scenario. The QR code image is taken as the original target (the image to be encrypted), the encrypted image is encoded according to the principle of sequential coding, and the position of each pixel before sequential coding is taken as the index matrix (private key 1) to complete the first-level encryption; Select the carrier image, hide the first-level encrypted image into the carrier image according to the SURF matching principle (private key 2), obtain the camouflage image, and complete the camouflage encryption. According to the ghost imaging algorithm, the camouflage image is modulated by the preset Hadamard matrix modulation mode (public key), and the ciphertext is obtained. The UAV receives ciphertext information through the detector, for non-users, the correct QR code image cannot be decrypted through the error key. Even if the eavesdropper gets the public key information, it can only decrypt the camouflage image, so it cannot land. For our users, after receiving the ciphertext, first decrypt the camouflage image according to the public key, then decrypt the carrier image and the first-level encrypted image according to the private key 2, and finally use the private key 1 for the final decryption of the first-level encrypted image to get the correct QR code image, so as to land. The feasibility and anti-noise attack performance of the algorithm are analyzed, and the encryption method can decrypt the image well and resist some noise attacks from the outside world, so it also has a high security.

## Theoretical basis

### QR code

**Figure 1:**
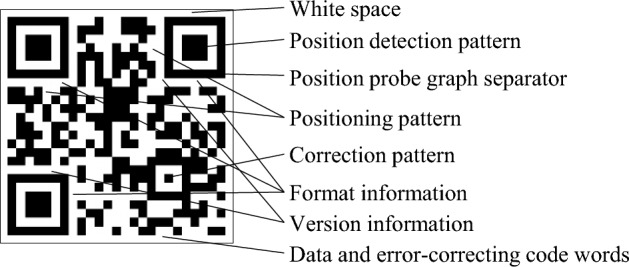
Diagram of QR code structure.

Two-dimensional code is a graphic distributed in two-dimensional space according to specific rules, which contains a number of information. The information is converted into the two-dimensional code format by encoding, and the two-dimensional code can be decoded to restore the contained information. There are many types of two-dimensional code, and in the UAV landing scenario, the most commonly used is QR code^27^. QR code is short for quick response and belongs to matrix 2D barcode^28^. The QR code is a square array, which is composed of a coding area and functional graphics including delimiters, positioning graphics, correction graphics and image finding graphics. Among them, functional graphics are not available for data encoding. The symbol is surrounded by a blank area, which has no actual coding and functional function. The QR code structure is shown in Fig. 1.

QR code has three characteristics: high data storage; Strong fault tolerance; Multi-angle high-speed recognition. There is a unique position detection graph on the three corners of the QR code to determine the specific position, direction and size of the QR code, so that the QR code has 360° rapid all-round reading ability.

### The principle of QR code encryption based on ghost imaging

**Figure 2:**
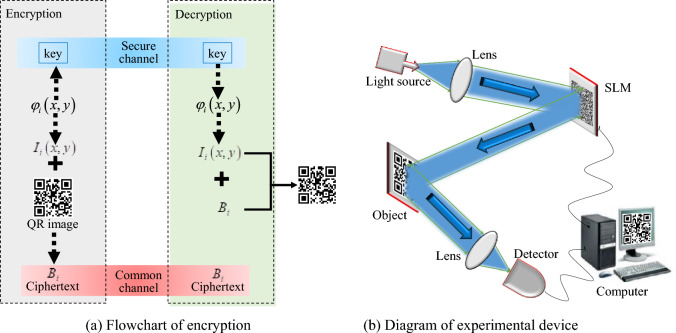
QR code encryption principle based on ghost imaging.

The specific process of QR code encryption based on ghost imaging is shown in Fig. 2a. The encrypter wants to transmit the encrypted QR code information to the decrypter. A series of random phase information $\varphi_{i} (x,y)(i = 1,{2}, \cdots ,N)$ is introduced into SLM by using the ghost imaging experimental device diagram shown in Fig. 2b. The phase value is evenly distributed between $\left[ {0,2\pi } \right]$. The light field $\left\{ {I_{i} \left( {x,y} \right)} \right\}$ generated by modulation is irradiated onto the object image, and the transmitted or reflected light is collected by the bucket detector. Here the QR code image is used as the plaintext object. The operation is repeated N times for N different phase information, and the obtained N bucket detector value $B_{i}$ is transmitted to the decrypter through the public channel as the ciphertext. The phase information $\varphi_{i} \left( {x,y} \right)\left( {N \times N} \right)$ is converted into a one-dimensional vector as a key $\varphi \left( {1 \times N^{2} } \right)$ and transmitted to the decrypter through secure channel.

1 $$ \begin{gathered} \varphi_{i} = \left[ {\begin{array}{*{20}c} {\varphi_{i} (1,1)} & {\varphi_{i} (1,2)} & \cdots & \cdots & {\varphi_{i} (1,y)} \\ {\varphi_{i} (2,1)} & {\varphi_{i} (2,2)} & \cdots & \cdots & {\varphi_{i} (2,y)} \\ \vdots & \vdots & \ddots & {} & \vdots \\ \vdots & \vdots & {} & \ddots & \vdots \\ {\varphi_{i} (x,1)} & {\varphi_{i} (x,2)} & \cdots & \cdots & {\varphi_{i} (x,y)} \\ \end{array} } \right] \\ \varphi = \left[ {\varphi_{i} (1,1) \cdots \varphi_{i} (1,y),\varphi_{i} (2,1) \cdots \varphi_{i} (2,y), \cdots ,\varphi_{i} (x,1) \cdots \varphi_{i} (x,y)} \right] \\ \end{gathered} $$

Whenever a phase message $\varphi_{i} \left( {x,y} \right)$ is loaded in SLM, the bucket detector obtains a detector value $B_{i}$. After receiving the transmitted ciphertext and key, the decrypter calculates the corresponding light field information $I_{i} \left( {x,y} \right)$ according to the Fresnel diffraction theorem for each phase information in the key:

2 $$ B_{i} = \int {I_{i} \left( {x,y} \right)T\left( {x,y} \right)dxdy} \, , \, I_{i} \left( {x,y} \right) = \left| {E_{in} \left( {x,y} \right)exp\left[ {j\varphi_{i} \left( {x,y} \right)} \right] \otimes h_{z} \left( {x,y} \right)} \right| $$

$h_{z} \left( {x,y} \right)$ is the Fresnel diffraction function propagated over a distance z, and $\otimes$ represents the convolution operation. According to the obtained light field information and ciphertext, the decrypter performs correlation calculation to obtain the plaintext information. The decryption process calculation formula is as follows:

3 $$ T_{GI} \left( {x,y} \right) = \frac{1}{N}\sum\limits_{i = 1}^{N} {\left( {B_{i} - \left\langle B \right\rangle } \right)I_{i} \left( {x,y} \right)} $$

$\left\langle B \right\rangle$ represents the average light intensity value and N represents the acquisition frequency.

## Double camouflage encryption method of QR code based on UAV landing scenario

**Figure 3:**
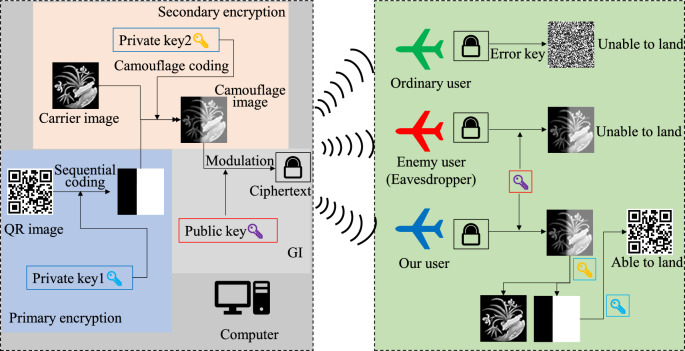
Double camouflage encryption principle of QR code in UAV landing scenario.

This paper proposes a double camouflage encryption method of QR code based on the UAV landing scenario, the principle of which is shown in Fig. 3. In this scheme, the QR code image needed for UAV landing is first double camouflage encrypted by private key and carrier image, and then modulated by public key according to the principle of ghost imaging to obtain ciphertext. After receiving the ciphertext, the receiver first decrypts the camouflage image according to the public key, and then decrypts the QR code image using the private key. The specific process includes two stages: encryption stage and decryption stage.

### Encryption stage

1. Taking the QR code image as the original target (image to be encrypted), the encrypted image is first encoded according to the sequential coding principle, which is first-level encryption, and the position of each pixel before the sequential encoding is used as the index matrix, that is, the private key 1. Since the image size of the QR code used in the experiment is $64 \times 64$, the matrix form of this private key is $$\left[ {\begin{array}{*{20}c} {QR(1,1)} & {QR(1,2)} & \cdots & \cdots & {QR(1,y)} \\ {QR(2,1)} & {QR(2,2)} & \cdots & \cdots & {QR(2,y)} \\ \vdots & \vdots & \ddots & {} & \vdots \\ \vdots & \vdots & {} & \ddots & \vdots \\ {QR(x,1)} & {QR(x,2)} & \cdots & \cdots & {QR(x,y)} \\ \end{array} } \right]$$. Where, $QR(i,j)$ represents the corresponding position of each pixel in the QR code image;
2. Select the carrier image, hide the first-level encrypted image into the carrier image according to the SURF matching principle, and obtain the camouflage image. This is second-level encryption, also known as camouflage encryption. This matching principle is used as the second-level key, namely the private key 2;
3. The camouflage image is modulated according to the preset modulation matrix mode to obtain ciphertext information. Here, a series of Hadamard matrices are used as the preset modulation mode, namely, the public key $H$, The process of generating the public key is $$H_{N} = H_{{2^{k} }} = H_{2} \bullet H_{{2^{k - 1} }} = \left[ {\begin{array}{*{20}c} {H_{{2^{k - 1} }} } & {H_{{2^{k - 1} }} } \\ {H_{{2^{k - 1} }} } & { - H_{{2^{k - 1} }} } \\ \end{array} } \right]$$. Where, $$H_{2} = \left[ {\begin{array}{*{20}c} 1 & 1 \\ 1 & { - 1} \\ \end{array} } \right]$$,$N = 2^{k} \left( {k = 1,2,3, \cdots } \right)$, $\bullet$ represents the Kronecker product.

### Decryption stage

(4) The receiver (UAV) receives the ciphertext image information through the detector. For ordinary users, under normal circumstances, it will not land if it recognizes the landing area that is not its own. Even if it is forced to decrypt, the correct QR code image cannot be decrypted through the wrong key, so it cannot land;
(5) For enemy users (eavesdroppers), the purpose is to crack the encryption system and get the correct key information. However, in this encryption scheme, even if the eavesdrover gets the public key information, it can only decrypt the camouflage image according to the ciphertext, and without the correct private key, it still cannot decrypt the position of the QR code, so it cannot land.
(6) For our users, after receiving the ciphertext information, first decrypt the camouflage image according to the public key, then decrypt the first-level encrypted image according to the private key 2, and then use the index matrix for the final decryption of the first-level encrypted image, get the correct QR code image, and finally land.

## Performance analysis

**Figure 4:**
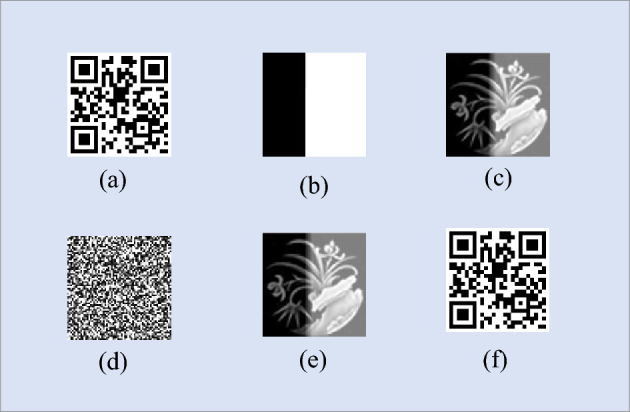
Algorithm simulation effect diagram: (**a**) The original image; (**b**) First encrypted image; (**c**) Camouflage image; (**d**) Error key decryption image(ordinary users); (**e**) Public key decryption image(enemy users); (**f**) Correctly decryption image(our users).

In the research process, this paper simulated the QR code image $\left( {64 \times 64} \right)$ under different sampling rates, and the decryption imaging effect through the encryption scheme is shown in Fig. 4. In order to fully analyze the scheme, the simulation is carried out from the two perspectives of feasibility and anti-noise attack performance. The peak signal-to-noise ratio is taken as the feasibility analysis index, and the anti-noise attack performance of the analysis scheme is verified by the two evaluation indexes of structural similarity and bit error rate.

The decryption effect of QR code image information of landing area by ordinary users, enemy users and our users is compared. For ordinary users, the correct QR code image cannot be decrypted through the wrong key, as shown in Fig. 4d,so it cannot land; For enemy users, even if the eavesdrover gets the public key information, it can only decrypt the camouflage image according to the ciphertext, and without the correct private key, it still cannot decrypt the position of the QR code, as shown in Fig. 4e, so it cannot land. For our users, after receiving the ciphertext information, first decrypt the camouflage image according to the public key, then use the private key for the final decryption, get the correct QR code image, as shown in Fig. 4f, and finally land.

### Feasibility analysis

In order to describe the accuracy of image recovery of the encryption algorithm in this paper, the Peak Signal-to-Noise Ratio (PSNR)^29^ is introduced here to measure the image decryption effect. For QR code images of pixel size $M \times N$, PSNR is defined as follows:

4 $$ MSE = \frac{1}{MN}\sum\limits_{{{\text{i}} = 1}}^{M} {\sum\limits_{j = 1}^{N} {\left( {X_{i,j} - X^{\prime}_{i,j} } \right)^{ \, 2} } } { ,}\,PSNR = 10\lg \frac{{X_{\max }^{2} }}{MSE}\left( {dB} \right) $$

where $X_{i,j}$ and $X_{i,j}{\prime}$ represent the size of the corresponding pixel values of the original image and the decrypted image respectively. $X_{\max }$ represents the largest pixel value in the image. In general, the higher the PSNR, the less distortion and the higher the recovery quality of the image.

**Figure 5:**
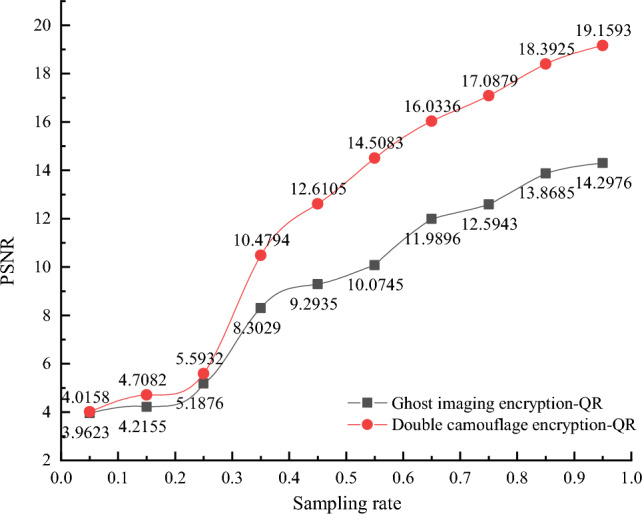
PSNR values of images decrypted by different algorithm at different sampling rates.

On the premise of correct key, the PSNR values of decrypted images obtained by the ghost imaging encryption algorithm based on QR code and the double camouflage encryption algorithm proposed in this paper are compared and analyzed, as shown in Fig. 5. Figure 5 shows the PSNR values of the decrypted images with the sampling rates of 5%, 15%, 25%, 35%, 45%, 55%, 65%, 75%, 85%, 95%, respectively. As can be seen from Fig. 5:(1)When the sampling rate is 25%, the PSNR value of the decrypted reconstructed image by the proposed algorithm is 5.5932; when the sampling rate is 55%, the PSNR value is 14.5083; and when the sampling rate is 85%, the PSNR value is 18.3925. It shows that with the increase of sampling rate, the clearer the decrypted image is, the larger the corresponding PSNR value is. The clarity of the decrypted image is proportional to the sampling rate.(2)When the sampling rate is 35%, the PSNR value of the decrypted reconstructed image by the proposed algorithm is 10.4794, while the PSNR value of the ghost imaging encryption algorithm is 8.3029; When the sampling rate is 75%, the PSNR of the reconstructed image decrypted by the proposed algorithm is 17.0879, while the PSNR of the ghost imaging encryption algorithm is 12.5943. In the same case, the proposed algorithm has higher resolution than the decrypted images obtained by the traditional ghost imaging algorithm.

Table 1 shows the decryption effect of the reconstructed image at the sampling rate of 5%, 15%, 25%, 35%, 45%, 55%, 65%, 75%, 85%, 95%. As can be seen from Table 1: (1) At the sampling rate $\le 35\%$, the QR code reconstructed by ghost imaging cannot be decrypted, because the resolution of the QR code is too low to be recognized and decoded. (2) At the sampling rate $\ge 35\%$, the algorithm can decrypt the QR code because it has certain error correction ability. When the sampling rate is 85% and 95%, the decoding effect of the reconstructed image is very much the same, that is, the decrypted image of the QR code is the original image without distortion, which is due to the strong error correction ability of the QR code. For the reconstructed QR effect under the condition of incomplete sampling rates of the ghost imaging, as long as the error correction ability of the QR code is not exceeded, the error correction algorithms can recover data to a certain extent.

**Table 1:**
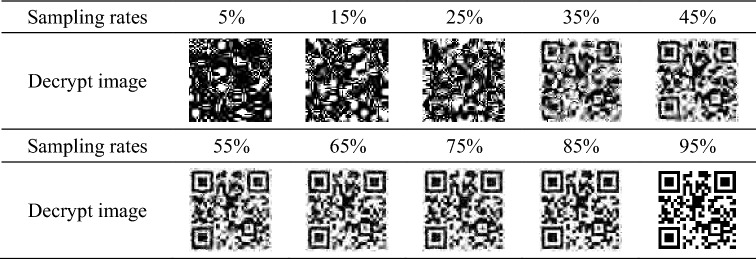
Decrypted QR code images at different sampling rates.

### Anti-noise attack

Noise attack is inevitable in the process of information transmission, and noise will affect the image quality and destroy the image information transmission. Here, pepper and salt noise (noise intensity 0.01, 0.02 and 0.04, respectively) is selected to simulate the attack. Objective evaluation index structure similarity (SSIM) and bit error rate (BER) are used to evaluate the anti-noise performance of the scheme. Structural similarity (SSIM) is a method to measure the similarity between the reconstructed image and the ideal image, which is consistent with the subjective perception of human eyes. The structure information is defined as brightness l, contrast c and structure attribute s. It is measured by mean $\mu_{x} ,\mu_{y}$, standard deviation $\delta_{x} ,\delta_{y}$ and covariance $\delta_{xy}$.$C_{1} ,C_{2} ,C_{3}$ represents a small positive number. The calculation formula is:

5 $$ \left\{ {\begin{array}{*{20}c} {\begin{array}{*{20}c} {l\left( {x,y} \right) = \frac{{2\mu_{x} \mu_{y} + C_{1} }}{{\mu_{x}^{2} + \mu_{y}^{2} + C_{1} }}} & {c\left( {x,y} \right) = \frac{{2\delta_{x} \delta_{y} + C_{2} }}{{\delta_{x}^{2} + \delta_{y}^{2} + C_{2} }}} & {s\left( {x,y} \right) = \frac{{\delta_{xy} + C_{3} }}{{\delta_{x} \delta_{y} + C_{3} }}} \\ \end{array} } \\ {\begin{array}{*{20}c} {\mu_{x} = \overline{x} = \frac{1}{N}\sum\nolimits_{i = 1}^{N} {x_{i} } } & {\mu_{y} = \overline{y} = \frac{1}{N}\sum\nolimits_{i = 1}^{N} {y_{i} } } & {\delta_{x} = \left( {\frac{1}{N - 1}\sum\nolimits_{i = 1}^{N} {\left( {x_{i} - \overline{x}} \right)^{2} } } \right)^{\frac{1}{2}} } \\ \end{array} } \\ {\begin{array}{*{20}c} {\delta_{y} = \left( {\frac{1}{N - 1}\sum\nolimits_{i = 1}^{N} {\left( {y_{i} - \overline{y}} \right)^{2} } } \right)^{\frac{1}{2}} } & {\delta_{xy} = \frac{1}{N - 1}\sum\nolimits_{i = 1}^{N} {\left( {x_{i} - \overline{x}} \right)\left( {y_{i} - \overline{y}} \right)} } \\ \end{array} } \\ {SSIM = l\left( {x,y} \right) \cdot c\left( {x,y} \right) \cdot s\left( {x,y} \right) = \frac{{\left( {2\mu_{x} \mu_{y} + C_{1} } \right)\left( {2\delta_{xy} + C_{2} } \right)}}{{\left( {\mu_{x}^{2} + \mu_{y}^{2} + C_{1} } \right)\left( {\delta_{x}^{2} + \delta_{y}^{2} + C_{2} } \right)}}} \\ \end{array} } \right. $$

In the process of image information transmission, due to the existence of various factors, errors often occur, resulting in error codes. The Bit Error Rate (BER) is generally expressed in scientific notation, and the lower the BER, the better. The calculation formula is shown in Eq. (6):

6 $$ BER = \frac{Error\, bit\, rate}{{Total\, bit\, rate}} \times 100\% $$

**Figure 6:**
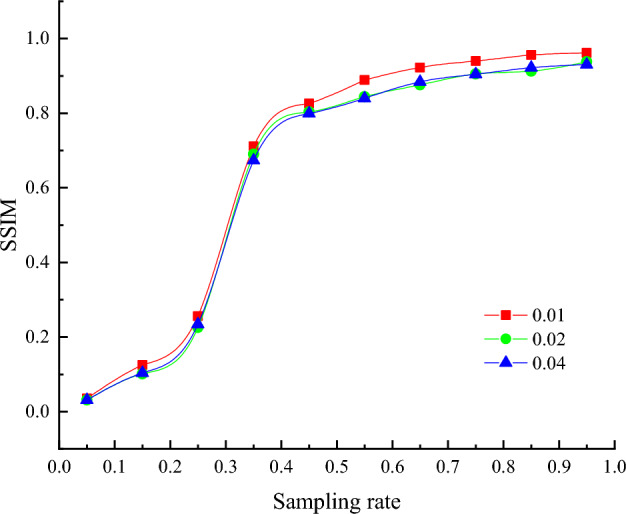
Changes of SSIM curves of QR code reconstruction under different intensity noise attacks.

Figure 6 and Table 2 show the change of SSIM curve and numerical comparison of the decryption imaging results of QR code image at different sampling rates in the process of simulated noise attack. In Fig. 6, red represents the noise attack intensity of 0.01, green represents the noise attack intensity of 0.02, and blue represents the noise attack intensity of 0.04. It can be seen from the curve change trend that: (1) With the increase of the sampling rate, the SSIM value becomes larger and larger, that is, the decryption image quality becomes closer and closer to the ideal image; (2) Under a certain intensity noise attack, when the sampling rate is 75%, 85%, 95%, the SSIM value of the decryption effect of the reconstructed image can reach above 0.9, indicating that the quality of the reconstructed image can be guaranteed.

**Table 2:** SSIM values reconstructed by QR code under different intensity noise attacks.

Intensity	5%	15%	25%	35%	45%	55%	65%	75%	85%	95%
0.01	0.0351	0.1246	0.2558	0.7106	0.8255	0.8885	0.9219	0.9398	0.9562	0.9616
0.02	0.03125	0.1009	0.2256	0.6898	0.8031	0.8438	0.8761	0.9054	0.9129	0.9375
0.03	0.0309	0.1035	0.2338	0.6736	0.7993	0.8401	0.8839	0.9045	0.9218	0.9305

**Figure 7:**
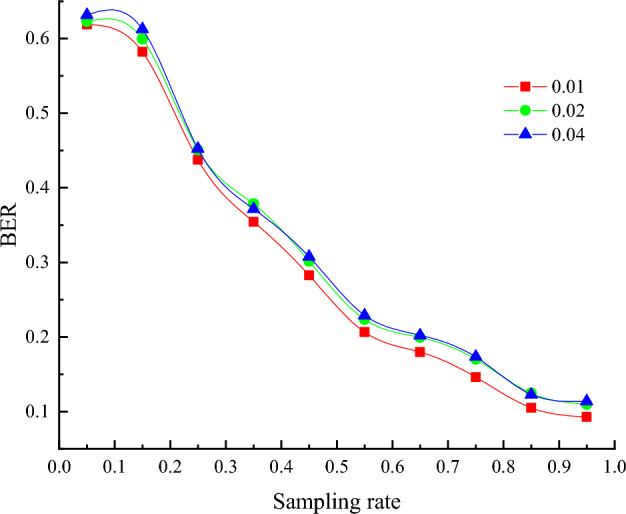
Changes of BER curves of QR code reconstruction under different intensity noise attacks.

Figure 7 and Table 3 show the change of BER value curve of the decryption imaging results of QR code images under different sampling rates in the process of simulated noise attack. In Fig. 7, red represents the noise attack intensity of 0.01, green represents the noise attack intensity of 0.02, and blue represents the noise attack intensity of 0.04. It can be seen from the curve change trend that: (1) With the increase of sampling rate, the BER value becomes smaller and smaller, that is, the decrypted image quality becomes higher and higher; (2) Under a certain intensity noise attack, the decryption imaging based on the encryption scheme in this paper can still guarantee a small enough BER value, which proves that the scheme has a good anti-noise attack ability.

**Table 3:** BER values reconstructed by QR code under different intensity noise attacks.

Intensity	5%	15%	25%	35%	45%	55%	65%	75%	85%	95%
0.01	0.6188	0.5821	0.4373	0.3542	0.2826	0.2065	0.1798	0.1462	0.1049	0.0928
0.02	0.6229	0.5994	0.4508	0.3782	0.3015	0.2236	0.1995	0.1702	0.1246	0.1098
0.03	0.6318	0.6126	0.4525	0.3715	0.3076	0.2289	0.2021	0.1735	0.1228	0.1139

## Conclusion

In the UAV landing scenario, the identification image of the landing point is very likely to be cracked by eavesdropping drones, so it is crucial to ensure the information security of the landing area and prevent it from being occupied by other users. Under normal circumstances, the landing area of the UAV is presented in QR code images, and the information security of the landing area can be ensured by using information encryption technology. In this paper, a double camouflage encryption method of QR code based on UAV landing scenario is proposed. The QR code image is taken as the original target, the encrypted image is encoded according to the sequential coding principle, and the first level encryption is completed, and the index matrix of the pixel before the sequential encoding is taken as the private key 1; Select the carrier image, and hide the first-level encrypted image into the carrier image according to the private key 2 to obtain the camouflage image and complete the camouflage encryption; According to the ghost imaging algorithm, the camouflage image is modulated by the preset Hadamard matrix modulation mode (public key), and the ciphertext is obtained. The drone receives the ciphertext information through the detector. For non-users, the correct QR code image cannot be decrypted through the error key. Even if the eavesdrover gets the public key information, it can only decrypt the camouflage image and cannot land. For our users, after receiving the ciphertext, first decrypt the camouflage image according to the public key, then decrypt the carrier image and the first-level encrypted image according to the private key 2, and finally use the private key 1 for the final decryption of the first-level encrypted image to get the correct QR code image, so as to land. In this paper, the feasibility and anti-noise attack performance of the algorithm are analyzed. The encryption method can decrypt the image well under the non-full sampling rate. Because the algorithm has double encryption effect and can resist certain external noise attacks, it also has high security.

## Data Availability

All data generated or analyzed during this study are included in this published article.

## References

[1] Gong WL, Zhao C, Yu H (2016). Three-dimensional ghost imaging lidar via sparsity constraint. Sci. Rep..

[2] Ye HL, Guo DD (2023). Research on mechanism of joint-coding imaging based on generative adversarial neural network. Opt. Lasers Eng..

[3] Kong LJ, Li Y, Qian SX (2013). Encryption of ghost imaging. Phys. Rev. A.

[4] Zhao SM, Wang L, Liang W (2015). High performance optical encryption based on computational ghost imaging with QR code and compressive sensing technique. Opt. Commun..

[5] Yuan S, Yao J, Liu X (2016). Cryptanalysis and security enhancement of optical cryptography based on computational ghost imaging. Opt. Commun..

[6] Sui LS, Du C, Xu MJ (2019). Information encryption based on the customized data container under the framework of computational ghost imaging. Opt. Express.

[7] Wu J, Xie Z, Liu Z (2016). Multiple-image encryption based on computational ghost imaging. Opt. Commun..

[8] Li X, Meng X, Yang X (2016). Multiple-image encryption based on compressive ghost imaging and coordinate sampling. IEEE Photonics J..

[9] Li X, Meng X, Yang X (2018). Multiple-image encryption via lifting wavelet transform and XOR operation based on compressive ghost imaging scheme. Opt. Lasers Eng..

[10] Yuan X, Zhang LH, Chen J (2019). Multiple-image encryption scheme based on ghost imaging of Hadamard matrix and spatial multiplexing. Appl. Phys. B.

[11] Du J, Xiong Y, Quan C (2020). High-efficiency optical image authentication scheme based on ghost imaging and block processing. Opt. Commun..

[12] Patra A, Saha A, Bhattacharya K (2022). Efficient storage and encryption of 32-slice CT scan images using phase grating. Arab. J. Sci. Eng..

[13] Yuan S, Han Y, Liu X (2023). Optical encryption for multi-user based on computational ghost imaging with Hadamard modulation. Optik..

[14] Yang Z, Yuan S, Li J (2022). An encryption method based on computational ghost imaging with chaotic mapping and DNA encoding. J. Opt..

[15] Luo CL, Guo F, Wan W (2022). Demonstration of ghost communication with an encrypted speckle. Opt. Laser Technol..

[16] Sun G, Huang WJ, Chen PF (2018). Advances in UAV-based Multispectral Remote Sensing Applications. Transactions of the Chinese Society for Agricultural Machinery..

[17] He Y, Li Z, Gao Z (2023). Autonomous and Precise Landing of uavs based on vision navigation. Electron. Opt. Control..

[18] Ye HL, Zhang LH, Zhang DW (2021). Non-imaging target recognition algorithm based on projection matrix and image euclidean distance by computational ghost imaging. Opt. Laser Technol..

[19] Xu X, Wang Z, Deng Y (2019). A software platform for vision based uav autonomous landing guidance based on markers estimation. Sci. China Technol. Sci..

[20] Gazzola, F., Marchini, E. A minimal time optimal control for a drone landing problem. *ESAIM: Control Opt. Calc. Var*. **27**, 99(2021).

[21] Monteiro M, Cecilia S, Cecilia C (2022). Simple physics behind the flight of a drone. Physics Education..

[22] Yang Y, Chen W, Zhu M (2020). Autonomous landing technology for drones based on machine vision. Foreign Electron. Measur. Technol..

[23] Lin, J., Bai, D., Gu, C. Design of a landing system for uav mobile platform based on machine vision. *Electron. World*. **7**,121–123 (2021).

[24] Mathivanan P, Balaji GA (2018). QR code based color image cryptography for the secured transmission of ECG signal. Multimed. Tools Appl..

[25] Mathivanan P, Balaji GA (2019). QR code-based ECG signal encryption/decryption algorithm. CRYPTOLOGIA..

[26] Mathivanan P, Balaji GA (2021). QR code based color image stego-crypto technique using dynamic bit replacement and logistic map. Optik..

[27] Wang JZ, Wang JF, Wang BC (2015). A security research on smart phone access control system based on QR code hybrid encryption technology. Netinfo Secur..

[28] Huang J (2013). The two-dimensional code QR code encoding of principle and realization. Comput. Knowl. Technol..

[29] Ye, H.L., Kang, Y., Wang, J., et al. A ghost imaging method based on multi-frequency fusion. *Eur. Phys. J. D*. **76**, 48 (2022).

